# Genome-wide association studies of smooth pursuit and antisaccade eye movements in psychotic disorders: findings from the B-SNIP study

**DOI:** 10.1038/tp.2017.210

**Published:** 2017-10-24

**Authors:** R Lencer, L J Mills, N Alliey-Rodriguez, R Shafee, A M Lee, J L Reilly, A Sprenger, J E McDowell, S A McCarroll, M S Keshavan, G D Pearlson, C A Tamminga, B A Clementz, E S Gershon, J A Sweeney, J R Bishop

**Affiliations:** 1 Department of Psychiatry and Psychotherapy, Otto Creutzfeldt Center for Cognitive and Behavioral Neuroscience, University of Muenster, Muenster, Germany; 2 Minnesota Supercomputing Institute, University of Minnesota, Minneapolis, MN, USA; 3 Department of Psychiatry and Behavioral Neuroscience, University of Chicago, Chicago, IL, USA; 4 Department of Genetics, Harvard Medical School, Boston, MA, USA; 5 Stanley Center for Psychiatric Research, Broad Institute of MIT and Harvard, Cambridge, MA, USA; 6 Department of Experimental and Clinical Pharmacology, College of Pharmacy, University of Minnesota, Minneapolis, MN, USA; 7 Department of Psychiatry and Behavioral Sciences, Northwestern University Feinberg School of Medicine, Chicago, IL, USA; 8 Department of Neurology, University of Luebeck, Luebeck, Germany; 9 Department of Psychology and Neuroscience, BioImaging Research Center, University of Georgia, Athens, GA, USA; 10 Department of Psychiatry, Harvard Medical School, Beth Israel Deacones Medical Center, Boston, MA, USA; 11 Departments of Psychiatry and Neurobiology, Yale University School of Medicine, New Haven, CT, USA; 12 Institute of Living, Hartford Hospital, Hartford, CT, USA; 13 Department of Psychiatry, University of Texas Southwestern Medical Center, Dallas, TX, USA; 14 Department of Psychiatry and Behavioral Neuroscience, University of Cincinnati, Cincinnati, OH, USA; 15 Department of Psychiatry, University of Minnesota College of Medicine, Minneapolis, MN, USA

## Abstract

Eye movement deviations, particularly deficits of initial sensorimotor processing and sustained pursuit maintenance, and antisaccade inhibition errors, are established intermediate phenotypes for psychotic disorders. We here studied eye movement measures of 849 participants from the Bipolar-Schizophrenia Network on Intermediate Phenotypes (B-SNIP) study (schizophrenia *N*=230, schizoaffective disorder *N*=155, psychotic bipolar disorder *N*=206 and healthy controls *N*=258) as quantitative phenotypes in relation to genetic data, while controlling for genetically derived ancestry measures, age and sex. A mixed-modeling genome-wide association studies approach was used including ~4.4 million genotypes (PsychChip and 1000 Genomes imputation). Across participants, sensorimotor processing at pursuit initiation was significantly associated with a single nucleotide polymorphism in *IPO8* (12p11.21, *P*=8 × 10^−11^), whereas suggestive associations with sustained pursuit maintenance were identified with SNPs in *SH3GL2* (9p22.2, *P*=3 × 10^−8^). In participants of predominantly African ancestry, sensorimotor processing was also significantly associated with SNPs in *PCDH12* (5q31.3, *P*=1.6 × 10^−10^), and suggestive associations were observed with *NRSN1* (6p22.3, *P*=5.4 × 10^−^^8^) and *LMO7* (13q22.2, *P*=7.3x10^−^^8^), whereas antisaccade error rate was significantly associated with a non-coding region at chromosome 7 (*P*=6.5 × 10^−9^). Exploratory pathway analyses revealed associations with nervous system development and function for 40 top genes with sensorimotor processing and pursuit maintenance (*P*=4.9 × 10^−^^2^–9.8 × 10^−4^). Our findings suggest novel patterns of genetic variation relevant for brain systems subserving eye movement control known to be impaired in psychotic disorders. They include genes involved in nuclear trafficking and gene silencing (*IPO8*), fast axonal guidance and synaptic specificity (*PCDH12*), transduction of nerve signals (*NRSN1*), retinal degeneration (*LMO7*), synaptic glutamate release (*SH3GL2*), and broader nervous system development and function.

## Introduction

Deviations of eye movement control are established neurophysiological intermediate phenotypes for psychotic disorders that may be useful for advancing gene discovery in psychiatry.^1^ Impairments are seen in a reduced ability to accurately track slowly moving objects with the eyes^2^ and to voluntarily suppress a reflexive saccade to a peripheral target on antisaccade tasks.^3, 4^ Consistent with multiple lines of evidence indicating shared neurobiological alterations and genetic vulnerability across schizophrenia spectrum and psychotic bipolar disorders,^5, 6, 7, 8, 9^ comparable eye movement deficits have been demonstrated across these groups in first-episode and chronically ill patients, and in their relatives indicating disturbances in brain systems subserving pursuit initiation and maintenance, and inhibitory control.^2, 10, 11, 12, 13, 14, 15, 16, 17, 18, 19, 20, 21, 22, 23, 24, 25, 26, 27^ We recently reported both smooth pursuit impairments and antisaccade inhibition errors in a large cohort of clinically stabilized psychotic disorder cases and their relatives as part of the Bipolar and Schizophrenia Network on Intermediate Phenotypes (B-SNIP) Consortium Study.^28, 29, 30^ We found that the initiation of a pursuit movement, which depends on rapid sensorimotor processing, was disturbed in probands and their relatives, while pursuit maintenance, dependent on cognitive predictions of target motion and the most widely used phenotype in prior genetic studies, was mostly impaired in probands.^29^ Impaired antisaccade task performance was identified in probands and their relatives, reflecting decreased inhibitory behavioral control.^28^ How these intermediate phenotypes are related to genetic variation across the genome has to date not been comprehensively studied.

The first genetic studies of eye movement abnormalities in psychotic disorders reported linkage between pursuit maintenance ability and microsatellite markers on the short arm of chromosome 6 (6p21-23).^31, 32^ Subsequent genetic studies using eye movement phenotypes have predominantly focused on single nucleotide polymorphisms (SNPs) in candidate genes for schizophrenia disease risk, for example, catechol-*O*-methyltransferase and neuregulin-1.^33, 34, 35, 36, 37, 38, 39^ Lencer *et al.*^40^ reported an association of pursuit-initiation impairments in first-episode psychosis patients with a dopamine D2 receptor gene (*DRD2*), whereas pursuit maintenance was associated to candidate SNPs in metabotropic glutamate receptor 3 protein (*GRM3).* This finding supports a model of different genes being potentially significant for different aspects of eye movement control.

Despite these initial reports, confirmation and larger scale genome-wide association studies (GWAS) in patients with psychosis are lacking. We report herein a GWAS evaluating genetic associations with three eye movement phenotypes representing (1) initial sensorimotor processing (pursuit acceleration), (2) sustained pursuit (maintenance gain) and (3) voluntary inhibitory control (antisaccade error rate) in probands with psychosis and controls from the B-SNIP sample, with additional exploratory pathway analyses to identify biological networks implicated by top findings.

## Materials and methods

### Participants

Smooth pursuit and antisaccade measures were assessed in 849 participants (schizophrenia *N*=230, schizoaffective disorder *N*=155, psychotic bipolar disorder *N*=206 and healthy controls *N*=258) of the B-SNIP consortium for whom DNA and genotyping information were available. In depth descriptions of the overall B-SNIP study design, inclusion and exclusion criteria, clinical ratings and eye movement assessments have been previously described.^28, 29, 30^ Diagnoses were made by a consensus process using all available clinical information including the Structured Clinical Interview for DSM IV (SCID)^41^ with collateral information from family members when available. Probands were clinically stable and receiving consistent psychopharmacological treatment for at least 1 month (Table 1; Supplementary Table 1).^42, 43, 44, 45, 46, 47^

Inclusion criteria for all subjects were (1) age 15–65; (2) WRAT reading score ⩾65;^42^ (3) no history of neurologic or systemic disease; (4) minimum of 20/40 visual acuity (with or without correction) and (5) no history of substance abuse within the last month or substance dependence within the last three months according to SCID, and negative urine toxicology (MP On-Site 11: One Step Onsite, ref: 60B02-MPB) on assessment day. Inclusion criteria for control subjects additionally included: (1) no personal or family history (first-degree) of psychotic or bipolar disorder; (2) no history of recurrent depression; and (3) no history of psychosis spectrum personality traits defined as meeting full or within one criteria of a cluster A (psychosis spectrum) Axis-II diagnosis. The study was approved by institutional review boards at each study site and written informed consent was obtained prior to study participation.

### Eye movement analyses

The eye movement measures (Table 1) utilized as primary outcome measures in genetic analyses included: (1) initial pursuit acceleration (measure of rapid sensorimotor processing during the first 100ms of pursuit assessed by foveo-petal step-ramp stimuli (18.7°/s);^29^ (2) pursuit maintenance gain (accuracy of matching eye to target velocity during sustained pursuit) using a triangular wave task (18.7°/s);^29^ and (3) antisaccade error rate defined as the percentage of trials with failed response inhibition from an overlap task,^28^ (Supplementary Material Methods). Eye movements were acquired with a video-based eye tracker in a darkened room (Eyelink II, SR Research, Ottawa, ON, Canada, sampling rate 500 Hz) with the same testing conditions and hardware used at all B-SNIP sites. Each eye movement measure was standardized using a normative regression approach, transforming data to *z*-scores including age, race and sex as covariates. This was done to remove variance in data related to demographic parameters from all groups in a similar way, and to facilitate comparison of the magnitude of effects across the different groups and pursuit measures. Our previous analyses with the B-SNIP study sample did not identify significant effects of antipsychotic dosing, anticholinergic loading or other medication effects on eye movement measures in these stably treated patients.^28, 29^ Furthermore, eye movement measures were shown to be relatively independent from general cognitive deficits indicated by BACS scores.^28, 29^

### Genotyping and imputation

Genomic DNA from participants was isolated from whole blood using standard protocols and genotyped by the Broad Institute using the Illumina Infinium PsychChip array. Quality control (QC) procedures were conducted with PLINK v1.9^48^ following standardized protocols.^49^ Genetic markers deviating from Hardy–Weinberg Equilibrium (*P*<10E−6), genotype-inferred sex differing from reported sex, or having call rates <98% were excluded from analyses. We included SNPs that had minor allele frequencies (MAF) ⩾0.01 in case or control groups. Cryptic relatedness was checked with PREST-plus.^50^ Samples showing a second degree relationship or greater were excluded resulting in 849 participants available for GWAS.

SNPs passing quality control procedures were used for imputation using HAPI-UR for pre-phasing^51^ and IMPUTE2 for imputation^52^ using the 1000 Genomes phase 1 data as a reference panel.^53^ Poorly imputed SNPs were filtered with the resulting imputed SNPs merged back in with the directly genotyped SNPs from the PsychChip for a total of 4 404 269 SNPs passing filtering criteria used for the analyses described herein.

Genetic ancestry assessments were completed with multi-dimensional scaling (MDS) plots relative to 1000 Genomes Project populations. Race stratified analyses represented a division of the two predominating ancestry components. Analyses of both stratified and whole group analyses utilized the first five principle components of ancestry analyses as covariates.

### Genome-wide association analyses approach

We used a mixed-modeling approach as implemented in the Efficient Mixed-Model Association eXpedited (EMMAX)-software package,^54^ which uses an identity by state (IBS) relationship matrix, and the first five eigenvectors from principle components analysis (PCA) included as covariates to reliably account for mixed ethnicity populations. Standardized eye movement measures (see above) were modeled as quantitative trait phenotypes in relation to genetic data. Probands and controls were grouped together for primary analyses with all ancestry groups combined. For secondary analyses, the sample was stratified by the top two genetically derived ancestry groups with follow-up studies in the proband only sample. The rationale for grouping cases and controls together in primary analyses was to take advantage of the wider range of phenotypic variance for the examination of genetic contributions to eye movement control.

To account for multiple testing using imputed data, the genome-wide significance threshold was set at 1 x E−08, which is more conservative than the commonly used GWAS significance threshold of 5 x E−08.^55^ False discovery rate (FDR) *q*-statistics further adjusting for multiple analyses of phenotypes and race groups were calculated. FDR *q*-values for GWAS significant findings remained <0.05 with the exception of rs2010148567 in relation to antisaccade response inhibition in African ancestry (AA), where *q*=0.09, all collectively indicating low type I error. We define ‘suggestive associations’ as *P*-values exceeding 5 x E−7 but not meeting 1 x E−8 GWAS significance. The closest gene was assigned to each SNP using BEDTools closest and RefSeq gene annotations from hg19.^56^

### Exploratory pathway analyses

We used Ingenuity Pathway Analysis software (Ingenuity Systems, Redwood City, CA, USA) to conduct exploratory analyses (using the Core Analysis feature) of genes affiliated (within 15 kb) with the top 200 SNP associations identified through GWAS analyses. Associations in all participants were examined separately for each eye movement measure by merging the top 200 associated SNPs from the two primary ancestry group analyses. The expression quantitative trait loci analyses of top SNPs associated with clinical phenotypes were performed using the Genotype-Tissue Expression GTEx Portal (www.gtexportal.org/home) and the United Kingdom Brain Expression Consortium (UKBEC, www.braineac.org).

## Results

### Initial sensorimotor processing

#### GWAS of initial pursuit acceleration in all participants

Across participants, the most robust genome-wide significant association was identified with an isolated SNP in the Importin 8 gene (*IPO8*) at chromosome 12p11.21 (Table 2). In addition, a number of SNPs in a non-coding RNA gene at chromosome 2p12, and in an intergenic region near the mitogen-activated protein kinase *MAP3K1* gene at chromosome 5q11.2 showed patterns of suggestive association.

#### GWAS of initial pursuit acceleration stratified by ancestry

**Figure 1:**
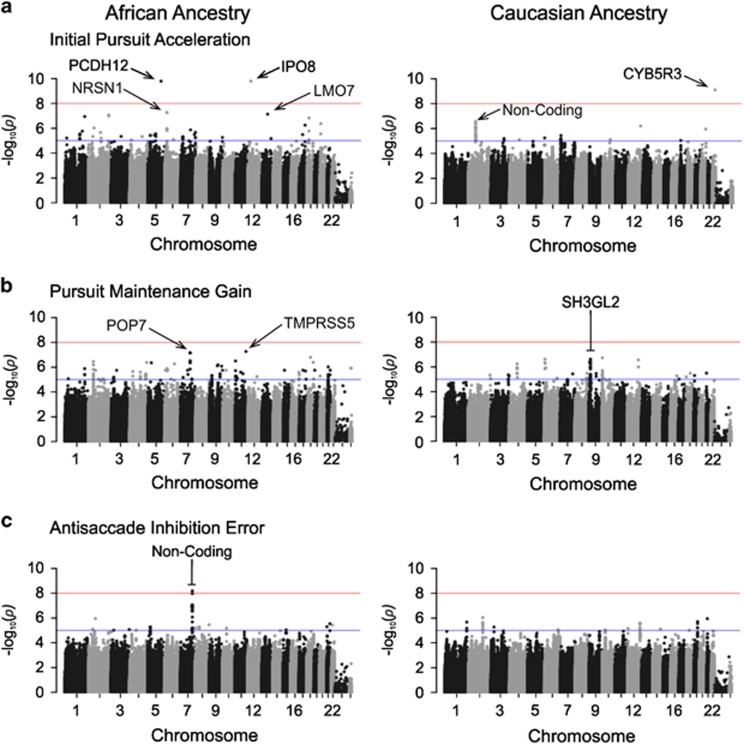
Manhattan plots from genome-wide association studies (GWAS) stratified for participants of predominantly African ancestry (*N*=300, left side) and participants of predominantly Caucasian ancestry (*N*=549, right side). Results for the three eye movement measures used as phenotypes in GWAS are depicted: (**a**) initial pursuit acceleration, (**b**) pursuit maintenance gain and (**c**) antisaccade error rate. For more details see Table 2.

GWAS in participants of predominantly AA (*N*=300) revealed the aforementioned genome-wide significant association with *IPO8*, as well as an additional genome-wide significant association with SNPs in the protocadherin 12 (*PCDH12*) gene at chromosome 5q31.3 (Figure 1a; Table 2). Other polymorphisms with suggestive associations included SNPs ~300 kb upstream of the Neurensin 1 gene (*NRSN1*) in an intergenic region at chromosome 6p22.3 and a SNP in the LIM domain only protein 7 gene (*LMO7*) at chromosome 13q22.2.

In participants of predominantly Caucasian ancestry (CA, *N*=549), initial pursuit acceleration was significantly associated with a SNP representing a missense mutation in *CYB5R3* at chromosome 22q13.2 coding for membrane bound cytochrome B5 reductase 3. In addition, the aforementioned SNPs in a non-coding RNA gene at chromosome 2p12 showed suggestive associations.

#### GWAS of initial pursuit acceleration in probands only

Follow-up analyses in probands across ancestries identified the genome-wide significant association with the SNP in *IPO8* that was seen in the whole-study sample, and additionally suggestive association in *LMO7.* Similarly, follow-up analyses in AA probands revealed the genome-wide significant associations with SNPs in *IPO8*, *PCDH12* and *LMO7* (Table 3). In the sub-sample of CA probands, no genome-wide significant association was observed with initial pursuit acceleration.

### Sustained pursuit maintenance

#### GWAS of maintenance gain in all participants

No associations were identified which exceeded our pre-defined threshold for genome-wide significance of 1 × E−8.^55^ Suggestive associations with pursuit maintenance gain across all participants were identified with a number of SNPs in or around the src Homology-3 (SH3) domain gene (*SH3GL2*) at chromosome 9p22.2 (Table 2).

#### GWAS of pursuit maintenance gain stratified by ancestry

Suggestive associations with *SH3GL2* were also observed in CA participants only (Table 2; Figure 1b). In AA participants, we identified further suggestive associations with pursuit maintenance ability. These included SNPs in *TMPRSS5* at chromosome 11q23.1 encoding a transmembrane serine protease, and in *POP7* at chromosome 7q22.1, which is a protein-coding gene related to gene expression and RNA transport. Very close to this region on chromosome 7, we additionally identified a SNP in *GGYF1,* which encodes a protein believed to act cooperatively with growth factor receptor-bound protein10 (*GRB10*) to regulate tyrosine kinase receptor signaling.

Follow-up analyses in the proband subsample (Table 3) showed suggestive associations of pursuit maintenance gain with SNPs in *POP7*, *GGYF1* and *TMPRSS5* in the subsample of AA probands but not in CA probands.

### GWAS of antisaccade error rates

GWAS with antisaccade error rate in AA participants identified one GWAS significant SNP and 25 suggestive SNPs in an intergenic region at chromosome 7 (Table 2; Figure 1c). However, no further associations with error rate were observed in either the whole sample or proband subsamples considered separately.

More details on top 200 SNPs identified in primary and secondary GWAS are given in Supplementary Table 2.

### Exploratory pathway analyses

**Figure 2:**
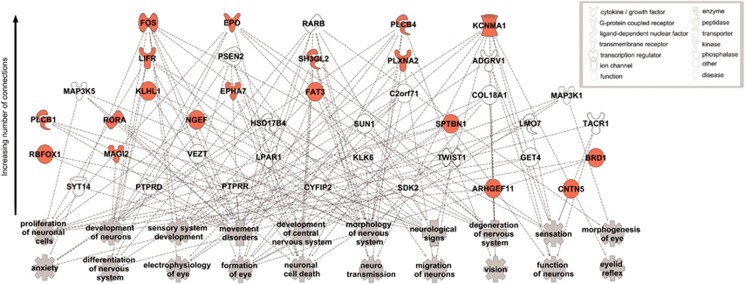
Summary of Ingenuity Pathway Analysis (IPA) using genes encoding the top 200 SNPs associated with initial pursuit acceleration and pursuit maintenance within the study population (*N*=849). The functional category nervous system development and function was identified as one of the top five physiological systems represented by these genes. Genes listed from the top have the greatest number of connections to functional categories within the nervous system development category listed in the lower panel. Genes that have shown evidence for a relationship to psychotic disorders are highlighted in red. SNP, single-nucleotide polymorphism.

The top 200 SNP associations identified in race stratified GWAS analyses represented 89 distinct genes for initial pursuit acceleration and 103 distinct genes for pursuit maintenance gain (Supplementary Table 3). A top physiological system category identified for both pursuit phenotypes was nervous system development and function, which was represented by ~19% (*N*=17) of the top genes associated with initial pursuit acceleration and ~22% (*N*=23) of the top genes associated with pursuit maintenance gain (enrichment *P*-value range 4.9−10^−2^–9.8 × 10^−4^). Noting the similarities in pathway relationships identified, the genes comprising these lists were merged (*N*=189 unique genes; *N*=40 genes relevant to the nervous system) and visualized with neural network mapping that highlights the nervous system development and synaptic functioning (Figure 2). This revealed functions including ‘formation of the eye’, ‘eyelid reflex’ and ‘electrophysiology of the eye’ ‘excitatory postsynaptic action potential’ as well as ‘neurological signs’, ‘movement disorders’ and ‘neurodegeneration’. Nineteen of these genes have previously shown evidence for a relationship to psychotic disorders.

## Discussion

In this GWAS, we focused on eye movement measures indexing different neurophysiological aspects of eye movement control known to be impaired in psychotic disorders. We identified novel genome-wide significant findings that may promote understanding of psychosis risk and pathophysiology. First, the most significant associations were found for initial sensorimotor processing with *IPO8* at 12p11.21, *PCDH12* at 5q31.3, *CYB5R3* at 22q13.2 and *LMO7* at 13q22.2. These associations were predominantly observed in variants with lower minor frequencies, mostly in AA participants. Second, suggestive associations with sustained pursuit maintenance were observed with protein coding SNPs in and around *SH3GL2* at 9p22.2. Third, significant genome-wide association of behavioral response inhibition was observed with a non-coding region at chromosome 7 in AA participants. All genes for which we found significant associations with referring SNPs are expressed in the brain (www.gtexportal.org/home; www.braineac.org). Those variants exceeding our pre-defined GWAS significance threshold also had low FDR statistics after accounting for multiple comparisons. Finally, exploratory pathway analyses of top associated SNPs identified commonalities between genes related to smooth pursuit measures, which consisted of loci previously associated with brain development, neurophysiology, ocular physiology and schizophrenia risk. These findings provide important new genetic information about what has long been one of the most promising familial phenotypes associated with psychotic disorders.^1, 2, 57^ This said, our findings extend previous reports from large-scale genetic studies showing considerable overlap between schizophrenia spectrum and bipolar disorders.^6, 7, 8, 9^

### Genetic alterations related to initial sensorimotor processing

The *IPO8* gene, in which we found a missense mutation significantly associated with initial pursuit acceleration, encodes importin 8, which has a key role in nuclear–cytoplasmic transport of proteins including many miRNAs.^58^ Importin 8 has also been identified as a component of miRNA-guided regulatory pathways for gene silencing by argonaute proteins, which are ubiquitous proteins found in plants, animals and fungi, leading to mRNA destabilization by transcription repression and translation inhibition.^59^ Blocking importin 8 reduces the nuclear concentration of argonaute proteins and may thus attenuate mRNA destabilization.^59^ We found this mutation, to date, only identified in those of AA, specifically associated with pursuit acceleration in AA probands.

Other suggestive associations with initial sensorimotor processing in the whole sample included a non-coding RNA gene (chr2p12), and SNPs ~15 kb upstream of *MAP3K1* (chr5q11.2), which encodes a mitogen-activated protein kinase known to regulate apoptosis.^60^ There were 52 other SNPs in or around *MAP3K1* including others upstream of the transcription starts site and two missense variants within the coding region, all with association *P*-values ranging from 3.4 × 10^−5^ to 2.2 × 10^−7^. In addition, expression quantitative trait loci analysis of the top associated SNP (rs1862618) revealed a strong correlation with the expression of the SET domain containing 9 gene (*SETD9*) (www.gtexportal.org/home) at chromosome 5q11.2, coding for a SET7 class of methyltransferase, which methylates H3K4. This correlation exists across multiple tissue types including regions of the brain and skeletal muscle.

Stratified GWAS by ancestry revealed significant genome-wide associations of sensorimotor processing with a synonymous mutation in *Protocadherin 12* (*PCDH12*) in AA participants, primarily driven by effects observed in AA probands. *PCDH12* belongs to a protocadherin gene cluster at chromosome 5q31 that has been previously implicated in schizophrenia and psychosis in non-AA samples.^61, 62^ *PCDH12* encodes a cellular adhesion molecule that has an important role in cell–cell interactions including axonal guidance and synaptic specificity. The association with initial pursuit acceleration suggests that in psychotic disorders alterations of the cadherin-based adhesive system may alter functional connectivity and coherent information processing in brain systems needed for fast visual information processing.^63^ Putative association of *PCDH12* with gyrification asymmetry has also been reported in schizophrenia suggesting its involvement in neurodevelopment and neural network formation.^64^ More broadly, our sensorimotor processsing related findings are in line with reports from the B-SNIP sample showing associations between genetic variants of the cadherin family and electroencephalogy abnormalities^65, 66^ and resting state brain activity seen with imaging studies.^67^

Suggestive associations of rapid sensorimotor processing around the Neurensin 1 gene (*NRSN1*, chr6p22.3) were observed in AA participants. *NRSN1* has been suggested to have an important role in the transduction of nerve signals and for neural plasticity. This may explain why *NRSN1* has been previously related to information-processing speed,^68^ supporting our finding of a specific association with rapid sensorimotor transformation needed during pursuit initiation.

Another genome-wide association was found for a missense mutation in the *LMO7* gene coding for LIM domain only protein 7 (chr13q22.2) in AA participants in general, and in AA probands specifically at a genome-wide significant level. *LMO7* is involved in protein–protein interactions and transcription, and mutations by alternative splicing in *LMO7* have been related to retinal defects and degeneration,^69^ which could explain why we found a SNP in this gene to be associated with rapid retinal error information processing.

Stratified GWAS in CA participants revealed genome-wide significant association of sensorimotor processing with *CYB5R3* (ch22q13.2). Notably, patients with 22q13 deletion syndrome are characterized by autism and schizophrenia-like symptoms.^70^ In these patients, loss of *CYB5R3* has been related to impaired language skills.^70^

### Genetic alterations related to pursuit maintenance

In contrast to pursuit initiation, sustained pursuit maintenance depends upon an established prediction of target velocity, and thus is more dependent on cognitive function. Here across all participants, we found suggestive associations of sustained pursuit maintenance with a region in the *SH3GL2* gene (chr9p22.2) encoding Endophilin A1.^71^ Previous studies in schizophrenia suggest that *SH3GL2* is differentially expressed in gray matter of prefrontal cortex in psychosis patients compared to controls.^72, 73^ Endophilin A1 is implicated in synaptic vesicle endocytosis involving intracellular signaling, calcium homeostasis and neurotransmitter release.^73^ Specifically, Endophilin A1 is suggested to regulate glutamate release in neurons expressing vesicular glutamate transporter 1.^74^ This is of interest as we recently found pursuit maintenance being associated with genetic variants in *GRM3*.^40, 75^

### Genetic alterations related to antisaccade response inhibition

SNPs associated with antisaccade performance in AA participants were identified in an intergenic region at chromosome 7 with the closest defined gene being the non-coding RNA LOC101928283, which is ~230 kb away. An expression quantitative trait loci analysis search for the top 10 SNPs within the United Kingdom Brain Expression Consortium (UKBEC) (www.braineac.org) showed significant association with expression of the gene *GPR37* (G protein-coupled receptor 37) within the hippocampus. The encoded protein contains seven transmembrane domains and is found in cell and endoplasmic reticulum membranes. G protein-coupled receptors are involved in translating outside signals into G protein-mediated intracellular effects. A previous GWAS on antisaccade error rate in twins reported suggestive associations with SNPs at chromosome 7 close to the region identified in the present study.^76^ The same study also revealed genome-wide significant associations with genes at chromosome 2.^76^ Others reported genome-wide linkage of antisaccade error rate with SNPs at chromosome 3p12 from a schizophrenia family study (COGS).^57^ Altogether, these findings support the notion that antisaccade error rate may be regarded as a complex polygenic trait.^76^

### Implications from pathway analyses

The 200 top SNPs associated with pursuit acceleration and maintenance gain were enriched for genes related to nervous system development pathways including relevant functions such as eye formation, neuronal action potential and movement disorders. Some of these genes have also been identified in previous disease risk association studies for psychotic disorders. Altogether, these findings support the model of smooth pursuit disturbances representing alterations in brain systems contributing to psychosis disease pathology. They are in line with other findings from the B-SNIP sample that revealed brain system changes related to gene clusters indicating physiological pathways involved in brain development, synaptic transmission and ion channel activity.^67, 77^

#### Limitations

Although our findings are novel and potentially heuristically valuable, there are potential limitations. First, although our sample size was large compared to most previous association studies of eye movements in psychosis probands, it is still small for GWAS. To enhance statistical power, we used a combined proband-control sample from the B-SNIP study, which had the benefit of increasing sample size as well as phenotypic variance for genetic association analyses. However, our analyses are not powered to detect smaller genotype–phenotype associations in the individual proband groups. Further research is needed to examine potential disorder-specific effects. Second, some of our more highly associated SNP findings represented those with lower minor allele frequencies (that is, *IPO8, PCDH12, CYB3R5, LMO7*). Special effort was undertaken to assure SNP genotyping quality and phenotyping for these variants, however these associations require validation and replication, especially with respect to the findings in the subgroup of AA participants.

## Conclusions

GWAS using eye movement phenotypes offers a promising approach for advancing pathophysiological models and understanding discrete components of complex multifactorial genetic risks for psychosis. We identified regions of interest for further study including some novel findings in addition to suggestive associations that are consistent with prior disease risk studies. Collectively, these findings highlight the importance of genes related to disease risk alongside other unique genetic contributions to eye movement phenotypes associated with psychotic disorders.

## Figures and Tables

**Table 1:** Characteristics of probands with psychosis and healthy controls

	*Psychosis probands,* N=*591*	*Healthy controls,* N=*258*	*Comparison*
Age, mean (s.d.)	35.4 (12.5)	37 (12.5)	NS
Sex (% male)	50%	46%	NS
Predominantly African Ancestry, n (%)^a^	224 (38%)	76 (29.5%)	*χ*^2^= 5.6; *P*=0.02
Predominantly Caucasian Ancestry, n (%)^a^	367 (62%)	182 (70.5%)	
WRAT 4 Word Reading^b^, mean (s.d.)	97.9 (15)	104.2 (13.7)	*t*_(842)_=5.8; *P*<0.001
BACS^c^ *z*-score, mean (s.d.)	−1.4 (1.4)	0.1 (1)	*t*_(847)_=16.1; *P*<0.001
PANSS^d^ positive, mean (s.d.)	15.9 (5.7)	NA	NA
PANSS^d^ negative, mean (s.d.)	14.8 (5.5)	NA	NA
PANSS^d^ total, mean (s.d.)	62.5 (17.3)	NA	NA
YMRS^e^, mean (s.d.)	6.1 (6.3)	NA	NA
MADRS^f^, mean (s.d.)	10.6 (9.2)	NA	NA
Chlorpromazine equivalents^g^, mean (s.d.)	467 mg (434.1)	NA	NA
Antidepressants, n (%)	273 (47%)	NA	NA
Mood stabilizer, n (%)	287 (49%)	NA	NA
			
*Smooth pursuit and antisaccade performance*			
Initial pursuit acceleration, mean (s.d.)	60.7°/s^2^ (34)	80.5°/s^2^ (35.7)	*t*_(847)_=8.56; *P*<0.001
Pursuit maintenance gain, mean (s.d.)	0.86 (0.17)	0.93 (0.1)	*t*_(835)_=7.64; *P*<0.001
Antisaccade error rate, mean (s.d.)	39.1% (26)	18.5% (13)	*t*_(137)_=4.34; *P*<0.001

Abbreviations: NS, not significant; NA, not applicable.

a According to principal component analyses (PCA).

b Wide Range Achievement Test 4th—Edition: Reading.^42^

c Brief Assessment of Cognition in Schizophrenia,^43^ *z*-scores are given relative to test norms.

d Positive and Negative Symptom Scale.^44^

e Montgomery Asberg Depression Rating Scale.^45^

f Young Mania Rating Scale.^46^

g According to Andreason *et al.*^47^

**Table 2:** Top SNPs associated with eye movement measures in the combined proband-control sample and in subsets defined by ancestry

*Cohort*	*Gene*	*SNP ID*	*Location*	*SNP type*	*Description*	P*-value*
*Initial pursuit acceleration*						
* *Combined ancestry	**IPO8**	**rs142754383**	**Chr12:30814184**	**Missense**	**c.1772A>G; K591R**	**7.8E−11**
	MAP3K1	rs1862618	Chr5:56096315	Intergenic	g.56096315G>C	2.15E−07
	LOC101927967	rs12617011	Chr2:77992269	Intergenic	g.77992269C>T	2.56E−07
	LOC101927967	rs2129493	Chr2:77990458	Intergenic	g.77990458A>T	2.9E−07
	LOC101927967	rs4853338	Chr2:77995848	Intergenic	g.77995848G>A	3.68E−07
	LOC101927967	rs1872787	Chr2:77990824	Intergenic	g.77990824A>G	3.74E−07
	LOC101927967	rs192238154	Chr2:77985105	Intergenic	g.77985105G>A	4.90E−07
	LOC101927967	rs2861081	Chr2:77991268	Intergenic	g.77991268C>T	4.91E−07
						
African ancestry	**IPO8**	**rs142754383**	**Chr12:30814184**	**Missense**	**c.1772A>G; K591R**	**1.61E−10**
	**PCDH12**	**rs105633**	**Chr5:141325249**	**Synonymous**	**c.3252A>G; P1084P**	**1.61E−10**
	NRSN1	rs144819560	Chr6:23818448	Intergenic	g.23818448A>G	5.35E−08
	NRSN1	rs76257869	Chr6:23755905	Intergenic	g.23755905T>C	5.44E−08
	LMO7	rs76082815	Chr13:76395342	Missense	c.2393C>T; P798L	7.34E−08
	SPAG16	rs72952023	Chr2:215177990	Intronic	c.1721-96874C>T	8.48E−08
	SPAG16	rs72952024	Chr2:215178086	Intronic	c.1721-96778G>A	1.02E−07
	LOC105372897	rs115777110	Chr1:209109556	Intergenic	g.209109556T>C	1.14E−07
	CABLES1	rs4800149	Chr18:20744254	Intronic	c.846-24548C>A	1.48E−07
	PLCB4	rs2299682	Chr20:9429344	Intronic	c.2844+4454A>G	4.27E−07
						
Caucasian ancestry	CYB5R3	rs61743746	Chr22:43015787	Missense	c.997G>A; V333I	7.68E−10
	LOC101927967	rs74261103	Chr2:77986915	Intergenic	g.77986915A>G	2.75E−07
	LOC101927967	rs13386612	Chr2:77987549	Intergenic	g.77987549 C>A	4.08E−07
	LOC101927967	rs10181488	Chr2:77987909	Intergenic	g.77987909T>G	4.08E−07
						
*Pursuit maintenance gain*						
Combined ancestry	SH3GL2	rs78314758	Chr9:17695593	Intronic	c.46-51471G>A	3.21E−08
	ACTL7A	rs56031956	Chr9:111625629	Missense	c.1027C>G; L343V	1.36E−07
	SH3GL2	rs145586720	Chr9:17693570-71	Intronic	c.46-53485_del-CA	1.40E−07
	SH3GL2	rs77484701	Chr9:17653768	Intronic	c.45+74483G>A	2.00E−07
	TTC16	rs77630455	Chr9:130487157	Missense	c.1240T>G; F414V	3.71E−07
	SH3GL2	rs16935877	Chr9:17687749	Intronic	c.46-59315G>A	3.73E−07
	UXS1	rs6738485	Chr2:106809960	Intronic	c.94+644G>A	3.79E−07
						
African ancestry	TMPRSS5	rs7939917	Chr11:113568096	Missense	c.373G>A, V125M	5.4E−08
	GIGYF1	rs221798	Chr7:100287495	Upstream	g.100287495C>G	6.89E−08
	POP7	rs221774	Chr7:100298984	Upstream	g.100298984A>G	6.96E−08
	POP7	rs221778	Chr7:100298024	Upstream	g.100298024A>G	7.07E−08
	MIR924HG	rs150177813	Chr18:37153343	Non-coding	g.37153343T>C	1.61E−07
	EPO	rs506597	Chr7:100313420	Intergenic	g.100313420A>G	3.02E−07
	C11orf21	rs188839109	Chr11:2323089	Start lost	c.3G>A; M1I	3.18E−07
	LOC730100	rs79125412	Chr2:52510092	Intronic	g.52510092G>T	3.49E−07
	CCDC102B	rs12052005	Chr18:66499548	Intronic	c.-15-4438G>T	4.07E−07
	POP7	rs2432929	Chr7:100299028	Upstream	g.100299028C>T	4.17E−07
	LINC01098	rs56196471	Chr4:179563815	Intergenic	g.179563815G>A	4.19E−07
	POP7	rs221770	Chr7:100302094	Upstream	g.100302094A>T	4.45E−07
	LIFR	rs3729734	Chr5:38527308	Missense	c.346C>T; H116Y	4.46E−07
						
** **Caucasian ancestry	AKR1C8P	rs139515701	Chr10:5219539	Intronic	c.93+7436C>G	1.85E−07
	SLC35B3	rs15300	Chr6:8413412	3′UTR	c.*370T>C	2.37E−07
	SH3GL2	rs16935877	Chr9:17687749	Intronic	c.46-59315G>A	2.38E−07
	KSR2	rs61945387	Chr12:118359414	Intronic	c.180+46467T>C	2.73E−07
	SH3GL2	rs145586720	Chr9:17693570-71	Intronic	c.46-53485_del-CA	3.32E−07
	PTPRD	rs12340173	Chr9:8346473	Intronic	c.4662-4495T>G	4.15E−07
	LOC100506207	rs9505461	Chr6:8495128	Non-coding	g.8495128C>G	4.55E−07
						
*Antisaccade error rate*						
** **African ancestry	**LOC101928283**	**rs201048567**	**Chr7:125255085-86**	**Intergenic**	**g.125255085-86_del-CA**	**6.45E−09**
	LOC101928283	rs34743817	Chr7:125255087-88	Intergenic	g.125255087-88_del-AT	1.06E−08
	LOC101928283	rs7781657	Chr7:125255150	Intergenic	g.125255150G>A	1.06E−08
	LOC101928283	rs12690985	Chr7:125258854	Intergenic	g.125258854G>T	9.25E−08
	LOC101928283	rs12706670	Chr7:125258919	Intergenic	g.125258919T>G	9.25E−08
	LOC101928283	rs12706671	Chr7:125259006	Intergenic	g.125259006A>C	9.25E−08
	LOC101928283	rs2402782	Chr7:125259452	Intergenic	g.125259452A>G	9.25E−08
	LOC101928283	rs1419699	Chr7:125261928	Intergenic	g.125261928G>T	9.25E−08
	LOC101928283	rs1579225	Chr7:125256488	Intergenic	g.125256488A>G	9.79E−08
	LOC101928283	rs1579226	Chr7:125256536	Intergenic	g.125256536G>A	9.79E−08
	LOC101928283	rs7785560	Chr7:125255700	Intergenic	g.125255700A>G	1.06E−07
	LOC101928283	rs7785979	Chr7:125255865	Intergenic	g.125255865C>T	1.06E−07
	LOC101928283	rs1579224	Chr7:125256395	Intergenic	g.125256395C>A	1.06E−07
	LOC101928283	rs6957945	Chr7:125256870	Intergenic	g.125256870A>T	1.06E−07
	LOC101928283	rs4634578	Chr7:125257232	Intergenic	g.125257232T>G	1.06E−07
	LOC101928283	rs10227132	Chr7:125258712	Intergenic	g.125258712G>A	1.06E−07
	LOC101928283	rs6467020	Chr7:125262711	Intergenic	g.125262711A>G	1.15E−07
	LOC101928283	rs6467021	Chr7:125262864	Intergenic	g.125262864G>A	1.15E−07
	LOC101928283	rs10234626	Chr7:125266178	Intergenic	g.125266178G>A	1.27E−07
	LOC101928283	rs10954078	Chr7:125265983	Intergenic	g.125265983G>A	1.37E−07
	LOC101928283	rs6958258	Chr7:125257092	Intergenic	g.125257092A>G	1.64E−07
	LOC101928283	rs4731257	Chr7:125266497	Intergenic	g.125266497A>G	2.32E−07
	LOC101928283	rs1579222	Chr7:125251771	Intergenic	g.125251771A>T	2.59E−07
	LOC101928283	rs6962819	Chr7:125257347	Intergenic	g.125257347G>A	2.80E−07

Listed are associations of *P*<5 × E−07, genome-wide significant associations (*P*<1 × E−08) are highlighted in bold.

**Table 3:** Top SNPs associated with eye movement measures across all probands and in subsets of probands defined by ancestry

*Cohort*	*Symbol*	*Assay ID*	*Location*	*SNP type*	*Description*	P*-value*
*Initial pursuit acceleration*						
* *Combined ancestry	IPO8	rs142754383	Chr12:30814184	Missense	c.1772A>G; K591R	9.0E−13
	LMO7	rs76082815	Chr13:76395342	Missense	c.2393C>T; P798L	3.99E−08
	SLC25A51P1	rs9354352	Chr6:66696272	Intergenic	g.66696272T>C	1.66E−07
	SLC25A51P1	rs7766730	Chr6:66697003	Intergenic	g.66697003C>A	3.67E−07
						
African ancestry	**PCDH12**	**rs105633**	**Chr5:141325249**	**Synonymous**	**c.3252A>G; P1084P**	**7.60E−12**
	**IPO8**	**rs142754383**	**Chr12:30814184**	**Missense**	**c.1772A>G; K591R**	**7.60E−12**
	**LMO7**	**rs76082815**	**Chr13:76395342**	**Missense**	**c.2393C>T; P798L**	**6.37E−09**
	STX2	rs137928907	Chr12:131311749	Missense	c.94T>G; F32V	2.33E−08
	RPN2	rs74417947	Chr20:35810114	Intronic	c.13+2342G>A	2.33E−08
	LOC105372897	rs115777110	Chr1:209109556	Intergenic	g.209109556T>C	2.33E−08
	ZNF740	rs74796725	Chr12:53581383	3′UTR	c.*9G>T	2.33E−08
	CABLES1	rs4800149	Chr18:20744254	Intronic	c.846-24548C>A	7.01E−08
	LTN1	rs57646126	Chr21:30331935	Missense	c.2438C>T; A813V	8.0E−08
	PLCB4	rs2299682	Chr20:9429344	Intronic	c.2844+4454A>G	9.44E−08
	SLC8A1-AS1	rs138449918	Chr2:40163950-1	Intronic	n.132+13922-3 del AT	1.07E−07
	ARL4C	rs13001243	Chr2:235214648	Intergenic	g.235214648G>A	2.06E−07
	ARL4C	rs35862416	Chr2:235212881	Intergenic	g.235212881G>A	2.06E−07
	ARL4C	rs36018891	Chr2:235214867	Intergenic	g.235214867T>C	2.06E−07
	ARL4C	rs71423631	Chr2:235213999	Intergenic	g.235213999A>G	2.06E−07
	MIR572	rs77867520	Chr4:11187849	Intergenic	g.11187849C>T	2.09E−07
	ARL4C	rs34115968	Chr2:235213474	Intergenic	g.235213474C>T	2.17E−07
	LOC285889	rs62482377	Chr7:156043640	Intergenic	g.156043640G>C	2.87E−07
	LOC285889	rs11523169	Chr7:156051554	Intergenic	g.156051554C>G	3.02E−07
	LOC285889	rs11523673	Chr7:156051784	Intergenic	g.156051784T>A	3.02E−07
	LOC285889	rs12698389	Chr7:156056275	Intergenic	g.156056275G>A	3.02E−07
	ARHGEF10L	rs146330533	Chr1:17996466	Intronic	c.2118+5376G>A	3.49E−07
	CABLES1	rs28625207	Chr18:20746672	Intronic	c.846-22130A>G	3.72E−07
	CCDC175	rs34486957	Chr14:60045597	5′ upstream	g.60045597C>T	4.27E−07
	LINC00615	rs75062117	Chr12:91277332	Intergenic	g.91277332G>A	4.89E−07
						
Caucasian ancestry	MIR5007	rs2997119	Chr13:56393900	Intergenic	g.56393900A>G	3.3E−07
						
*Pursuit maintenance gain*						
Combined ancestry	UXS1	rs6738485	Chr2:106809960	Intronic	c.94+644G>A	3.32E−07
	ACTL7A	rs56031956	Chr9:111625629	Missense	c.1027C>G; L343V	3.78E−07
						
African ancestry	POP7	rs221774	Chr7:100298984	Upstream	g.100298984A>G	1.01E−08
	GIGYF1	rs221798	Chr7:100287495	Upstream	g.100287495C>G	1.01E−08
	POP7	rs221778	Chr7:100298024	Upstream	g.100298024A>G	1.15E−08
	C11orf21	rs188839109	Chr11:2323089	Start lost	c.3G>A; M1I	2.33E−08
	LINC01098	rs56196471	Chr4:179563815	Intergenic	g.179563815G>A	8.68E−08
	EPO	rs506597	Chr7:100313420	Intergenic	g.100313420A>G	1.01E−07
	POP7	rs2432929	Chr7:100299028	Upstream	g.100299028C>T	1.26E−07
	POP7	rs221770	Chr7:100302094	Upstream	g.100302094A>T	1.38E−07
	CCDC102B	rs12052005	Chr18:66499548	Intronic	c.-15-4438G>T	2.62E−07
	LOC100506422	rs2571521	Chr9:26133808	Intergenic	g.26133808C>G	3.08E−07
	TMPRSS5	rs7939917	Chr11:113568096	Missense	c.373G>A, V125M	3.96E−07
						
Caucasian ancestry	KSR2	rs61945387	Chr12:118359414	Intronic	c.180+46467T>C	2.32E−07
	KSR2	rs17511946	Chr12:118353809	Intronic	c.180+52072T>C	4.20E−07
						
*Antisaccade error rate*						
African ancestry	LOC101929645	rs679895	Chr5:29091685	Intergenic	g.29091685C>T	2.24E−07
	LOC101929645	rs251058	Chr5:29093971	Intergenic	g.29093971T>A	2.25E−07
	ATP6V1E2	rs11125080	Chr2:46732405	Intronic	n.776-14435C>T	2.37E−07
	LOC101928283	rs201048567	Chr7:125255085-86	Intergenic	g.125255085-86_del-CA	2.45E−07
	LOC101929645	rs185168	Chr5:29093926	Intergenic	g.29093926C>T	2.49E−07
	LOC101928283	rs34743817	Chr7:125255087-88	Intergenic	g.125255087-88_del-AT	4.33E−07
	LOC101928283	rs7781657	Chr7:125255150	Intergenic	g.125255150G>A	4.33E−07
	LOC101929645	rs160309	Chr5:29100077	Intergenic	g.29100077T>A	4.33E−07
	LOC101929645	rs168759	Chr5:29095301	Intergenic	g.29095301G>A	4.33E−07
	LOC101929645	rs170138	Chr5:29098460	Intergenic	g.29098460A>T	4.33E−07
	LOC101929645	rs193967	Chr5:29095648	Intergenic	g.29095648G>A	4.33E−07
	LOC101929645	rs309675	Chr5:29107238	Intergenic	g.29107238G>T	4.33E−07
	LOC101929660	rs309677	Chr5:29109039	Intergenic	g.29109039T>G	4.33E−07
	LOC101929660	rs309678	Chr5:29109286	Intergenic	g.29109286G>C	4.33E−07
	LOC101929660	rs160312	Chr5:29112050	Intergenic	g.29112050T>C	4.42E−07

Abbreviation: SNP, single-nucleotide polymorphism. Listed are associations of *P*<5 × E−07, genome-wide significant associations (*P*<1 × E−08) are highlighted in bold.
