# Supplementary material for: Genome-wide association studies of smooth pursuit and antisaccade eye movements in psychotic disorders: findings from the B-SNIP study
Source: Transl Psychiatry. 2017 Oct 24;7(10):e1249–. doi: 10.1038/tp.2017.210 (PMC5682604; doi:10.1038/tp.2017.210)
Supplement: Supplementary Material Methods [file tp2017210x1.docx]

**Supplementary material - Methods**

**Eye movement task details**

Before testing, participants refrained from nicotine or caffeine exposure for at least 30 minutes. They were seated 60 cm from a 22-inch CRT monitor (1360 x 768 resolution; 150 Hz refresh rate) with their heads stabilized with a chin and forehead restraint. The target was a red cross in a box covering 0.5°. To assess initial eye acceleration during pursuit 32 foveo-petal step-ramps starting from central position were presented.[^1^](#_ENREF_1) Step size was 2.4° to either right or left appearing in randomized order followed immediately by a target sweep moving at a constant velocity of 18.7°/s in the opposite direction in horizontal plane. Step size was defined in a way that the target crossed the central position after 133ms, close to the time of pursuit initiation, without eliciting an initial catch-up saccade. Initial eye acceleration was computed by linear regression (RobustFit® in MatLab) of eye velocity in a 100ms time window beginning after the eyes had started to pursue the target.[^2^](#_ENREF_2) To assess sustained pursuit maintenance we used a triangular waveform with target sweeps, also at a constant velocity of 18.7°/s in the horizontal plane (+/- 12°). Forty-eight sweeps were used for analyses. Additionally, blocks of either 9.7°/s or 26.6°/s sweeps (30% of trials) were interspersed occasionally to enhance engagement but were not included in analyses. Median eye velocity was determined in intervals 300 to 840ms after reversal of target direction and related to target velocity in that same interval giving maintenance pursuit gain. Calibration trials were presented between blocks of trials for offline recalibration.

The task to assess antisaccade performance consisted of 80 overlap trials divided into four blocks. After a period of central fixation (1500 – 2500 msec), the central target was extinguished 200ms after peripheral target appearance at either 10° or 15° from center. Subjects were instructed to not look to the peripheral target (antisaccade error) but instead immediately look to the mirror image location in the opposite hemi-field. We determined the percentage of trials with antisaccade errors indicating failed inhibitory control.

1. Rashbass C. The relationship between saccadic and smooth tracking eye movements. *J Physiol* 1961; **159:** 326-338.

2. Carl JR, Gellman RS. Human smooth pursuit: stimulus-dependent responses. *J Neurophysiol* 1987; **57**(5)**:** 1446-1463.
