# Supplementary material for: Genome-wide association studies of smooth pursuit and antisaccade eye movements in psychotic disorders: findings from the B-SNIP study
Source: Transl Psychiatry. 2017 Oct 24;7(10):e1249–. doi: 10.1038/tp.2017.210 (PMC5682604; doi:10.1038/tp.2017.210)
Supplement: Supplementary Figure 1 [file tp2017210x2.pdf]

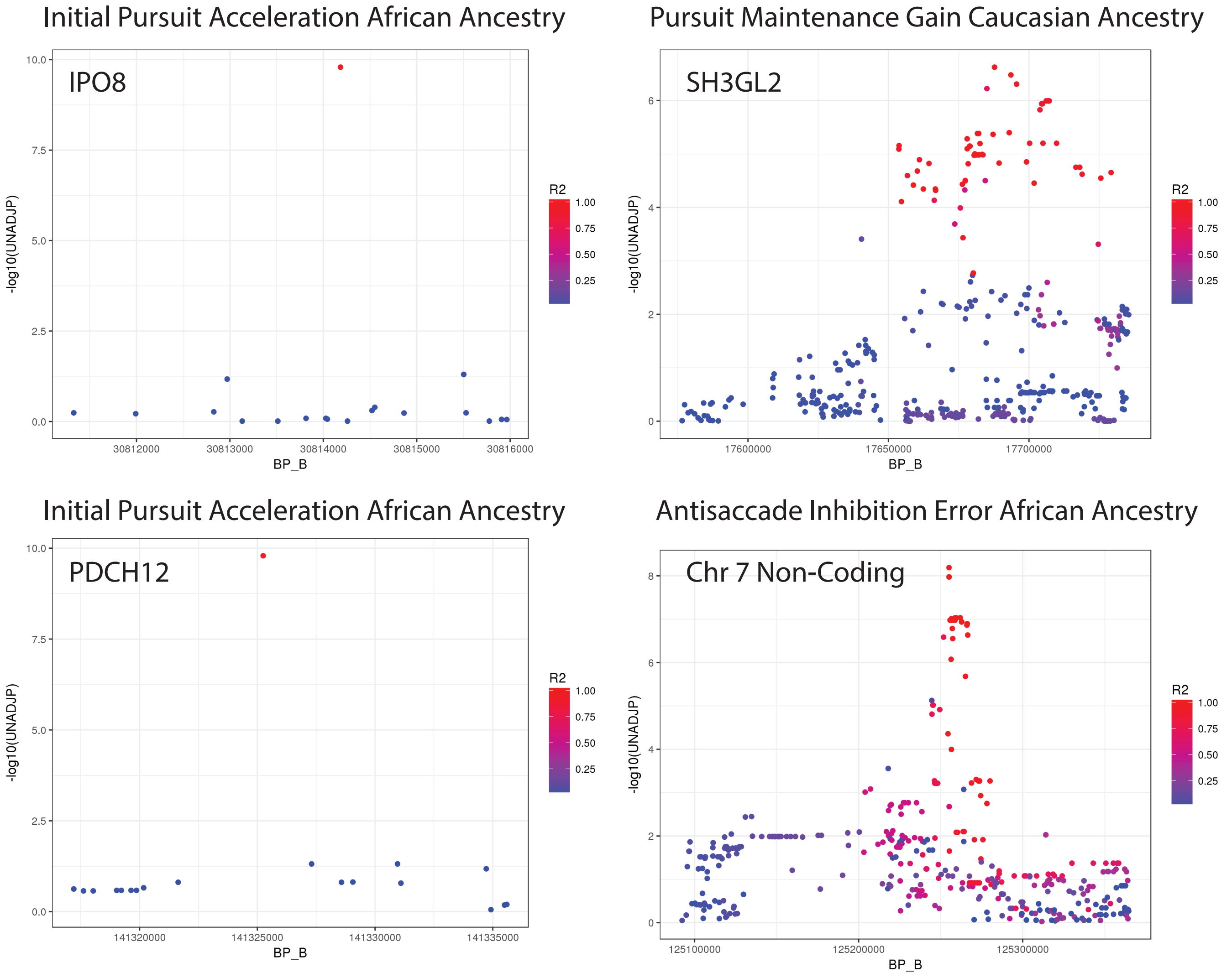
Initial Pursuit Acceleration African Ancestry
IPO8
-log10(UNADJP)
10.0
7.5
5.0
2.5
0.0
30812000
30813000
30814000
30815000
30816000
BP_B
R2
1.00
0.75
0.50
0.25
Pursuit Maintenance Gain Caucasian Ancestry
SH3GL2
-log10(UNADJP)
6
4
2
0
17600000
17650000
17700000
BP_B
R2
1.00
0.75
0.50
0.25
Initial Pursuit Acceleration African Ancestry
PDCH12
-log10(UNADJP)
10.0
7.5
5.0
2.5
0.0
141320000
141325000
141330000
141335000
BP_B
R2
1.00
0.75
0.50
0.25
Antisaccade Inhibition Error African Ancestry
Chr 7 Non-Coding
-log10(UNADJP)
8
6
4
2
0
125100000
125200000
125300000
BP_B
R2
1.00
0.75
0.50
0.25
