# Supplementary material for: Genome-wide association studies of smooth pursuit and antisaccade eye movements in psychotic disorders: findings from the B-SNIP study
Source: Transl Psychiatry. 2017 Oct 24;7(10):e1249–. doi: 10.1038/tp.2017.210 (PMC5682604; doi:10.1038/tp.2017.210)
Supplement: Supplementary Table 1 [file tp2017210x3.doc]

**Supplementary Table 1. Characteristics in Proband Subsamples**

|  | **PROBANDS** | | |
| --- | --- | --- | --- |
|  | **Schizo-phrenia**  **N=230** | **Schizo-affective**  **N=155** | **Bipolar Disorder**  **N=206** |
| Age, Mean (SD) | 34.5 (12.7) | 35.8 (11.8) | 34.2 (11.9) |
| Sex (% Male) | 67% | 40% | 36% |
| Predominantly African Ancestry^a^ | 114 (49.6%) | 69 (44.5%) | 41 (19.9%) |
| Predominantly Caucasian Ancestry^a^ | 116 (50.4%) | 86 (55.5%) | 165 (80.1%) |
| WRAT 4 Word Reading^b^, Mean (SD) | 94.1 (15.9) | 96.8 (14.0) | 100.5 (13.3) |
| BACS z-scores^c^, Mean (SD) | -1.8 (1.4) | -1.6 (1.3) | -1.1 (1.3) |
| PANSS Positive^d^, Mean (SD) | 17.1 (5.7) | 18.4 (4.9) | 12.7 (4.4) |
| PANSS Negative^d^, Mean (SD) | 16.3 (5.8) | 16.5 (5.0) | 12.2 (4.2) |
| PANSS Total^d^, Mean (SD) | 65.9 (17.5) | 70.3 (15.4) | 53.4 (13.4) |
| YMRS^e^, Mean (SD) | 5.6 (6.1) | 7.4 (6.1) | 4.8 (6.2) |
| MADRS^f^, Mean (SD) | 8.5 (8.3) | 13.9 (10.1) | 9.2 (8.9) |
| **Medication status** |  |  |  |
| Chlorpromazine Equivalents^g^, Mean (SD) | 529mg (445) | 516mg (530) | 348mg (320) |
| Antidepressants, N (%) | 40% | 57% | 47% |
| Mood Stabilizer, N (%) | 24% | 55% | 74% |
| **Smooth Pursuit and Antisaccade Performance** | | | |
| Initial Eye Acceleration, Mean (SD) | 57°/s^2^ (38.6) | 57.8°/s^2^ (30.4) | 59.6°/s^2^ (29.5) |
| Maintenance Pursuit Gain, Mean (SD) | 0.82 (0.18) | 0.87 (0.15) | 0.84 (0.18) |
| Antisaccade Error rate, Mean (SD) | 44% (27) | 37% (23) | 35% (24) |

^a^according to principal component analyses (PCA); ^b^Wide Range Achievement Test 4^th^ - Edition: Reading^40^; ^c^Brief Assessment of Cognition in Schizophrenia^41^, z-scores are given relative to test norms; ^d^Positive and Negative Symptom Scale^42^; ^e^Montgomery Asberg Depression Rating Scale^43^; ^f^Young Mania Rating Scale^44^; ^g^According to Andreason et al. (2010)^45^
