# Supplementary material for: Genome-wide association studies of smooth pursuit and antisaccade eye movements in psychotic disorders: findings from the B-SNIP study
Source: Transl Psychiatry. 2017 Oct 24;7(10):e1249–. doi: 10.1038/tp.2017.210 (PMC5682604; doi:10.1038/tp.2017.210)
Supplement: Supplementary Table 2 and 3 legends [file tp2017210x6.docx]

Supplementary Table 2. Top 200 SNP associations with eye movement phenotypes

Legend. Top 200 SNP associations with smooth pursuit acceleration (accel), gain (gain), and antisaccade error rates (anti) are presented in separate tabs for all participants (All), predominantly Caucasian ancestry (CA), and predominantly African ancestry (AA).

Supplementary Table 3. Gene lists for Ingenuity Pathway Analysis
